# *TGF-β1* Gene -509C/T Polymorphism and Coronary Artery Disease: An Updated Meta-Analysis Involving 11,701 Subjects

**DOI:** 10.3389/fphys.2017.00108

**Published:** 2017-02-23

**Authors:** Yan-yan Li, Yan-hong Zhou, Ge Gong, Hong-yu Geng, Xin-xing Yang

**Affiliations:** Department of geriatrics, First Affiliated Hospital of Nanjing Medical UniversityNanjing, China

**Keywords:** *TGF-β1*, -509C/T, polymorphism, coronary artery disease

## Abstract

**Background:** The *transforming growth factor-β1 (TGF-β1)* gene -509C/T polymorphism has been suggested to be associated with increased coronary artery disease (CAD) risk. However, the individual studies results are still inconsistent.

**Objective and methods:** To investigate the relationship between *TGF-β1* gene -509C/T polymorphism and CAD, a meta-analysis involving 11,701 participants from 8 individual studies was conducted. The pooled odds ratios (ORs) and their corresponding 95% confidence intervals were evaluated by using random or fixed effect models.

**Results:** A significant association between *TGF-β1* gene -509C/T polymorphism and CAD was detected in the total population under allelic (OR: 1.130, 95% CI: 1.060–1.200, *P* = 0.0001), recessive (OR: 1.390, 95% CI: 1.100–1.750, *P* = 0.006), dominant (OR: 0.857, 95% CI: 0.785–0.935, *P* = 2.507 × 10^−4^), homozygous (OR: 1.258, 95% CI: 1.098–1.442, *P* = 0.001), heterozygous (OR: 1.147, 95% CI: 1.046–1.257, *P* = 0.003), and additive genetic models (OR: 1.131, 95% CI: 1.063–1.204, *P* = 5.442 × 10^−5^). In the subgroup analysis, there was a significant association between them in Chinese population under all of the genetic models (*P* < 0.05), except under the heterozygous genetic model (*P* > 0.05). In the Caucasian subgroup, a significant association between them was also detected under all of the genetic models (*P* < 0.05), except under the recessive genetic model (*P* > 0.05).

**Conclusions:** *TGF-β1* gene -509C/T polymorphism was significantly associated with increased CAD risk. The people with T allele of *TGF-β1* gene -509C/T polymorphism might be predisposed to CAD.

## Introduction

The coronary artery disease (CAD) morbidity rose markedly which presented a rejumvanation trend (Zou et al., 2013). According to the mortality and disability rate, CAD is the most important disease in the United States of America (USA) and other industrialized countries. In the nearly one million patients who died of the cardiovascular disease in USA every year, CAD accounted for 50%. About 1.5 million USA patients were attacked by acute myocardial infarction which was almost caused by coronary atherosclerosis (Yang et al., 2006). CAD is the myocardial ischemia or necrosis disease caused by the artery lumen stenosis due to the coronary atherosclerosis. The CAD pathogenesis might be the combined actions result by heredity and environmental factors. The single nucleotide polymorphism is the important genetic factor which influences the CAD susceptibility.

The human *transforming growth factor-β1* (*TGF-β1*) gene, located in 19q13 chromosome, spans 2.5 kb which consists of 7 exons and 6 introns. The researches have confirmed that TGF-β1 could increase the extracellular matrix, inhibit the vascular smooth muscle cells proliferation, promote the vascular endothelial cells, and regulate the local inflammation. Thus TGF-β1 could influence the CAD progress (Aihara et al., 2010). The -509C/T (rs1800469) mutation, located in the upstream of the *TGF-β1* gene transcriptional initiation site, is the negative regulation region. C/T mutation alters the affinity of the promoter and transcription factor which cause the reduced TGF-β1 transcription level and participate in the CAD progression (Yokota et al., 2000).

Although many studies on the relationship between *TGF-β1* gene -509C/T polymorphism and CAD have been performed so far, the individual studies results were still conflicting. In 2014, Pei et al. found the TT genotype of the *TGF-β1* gene -509C/T polymorphism increased the CAD risk in a Chinese population (Pei et al., 2014). In addition, in another Chinese population Zou et al. also got the similar conclusion in 2013 (Zou et al., 2013). On the contrary, in 2006, Koch et al. did not find any significant genotype distributions difference of *TGF-β1* gene -509C/T gene polymorphism between control and CAD patients and they concluded that the *TGF-β1* gene -509C/T polymorphism was not associated with CAD in a Caucasian population (Koch et al., 2006). In 2009, Drenos got the similar negative conclusion in another Caucasian population (Drenos et al., 2009).

In the current study, a meta-analysis involving 6,377 CAD patients and 5,324 controls from eight separate studies was performed (Supplement S1). The current meta-analysis will help us further estimate the relationship of *TGF-β1* gene -509C/T polymorphism and CAD and formulate a novel individualized therapy strategy of CAD.

## Materials and methods

### Publication search and inclusion criteria

The terms as “*TGF-β1*,” “-509C/T,” “polymorphism” and “coronary artery disease” were used to retrieve the electronic databases including China Biological Medicine Database, China National Knowledge Infrastructure, PubMed, Embase, and Web of Science. The last research was updated on December 21, 2016 with the publication years ranging from 1996 to 2014.

The recruited studies should conform to such major criteria as the follows: (a) Estimation of the association of *TGF-β1* gene -509C/T polymorphism and CAD. (b) CAD was diagnosed by coronary angiography and the criteria was defined as the luminal narrowing in at least one coronary artery was no less than 50%. The patients with any previous history of documented CAD, with or without coronary revascularization were also classified to be CAD group (Li et al., 2016). The healthy population in the similar period without coronary artery stenosis was included in the control group. (c) The case-control or cohort studies published officially should be included. (d) The control group genotype member should follow the Hardy-Weinberg equilibrium (HWE).

### Data extraction

According to a standard protocol, the data was drawn out. The meta-analysis was performed by three investigators; two of whom were responsible for searching out the individual studies in duplicate, and the third one was to resolve the disputes between the two investigators. Those studies that did not meet the major selection criteria, that were repeated publications, or that supplied inadequate data were got rid of the present meta-analysis. If similar data was found in different papers by the same author group, the data was only adopted for one time. The following items as publication year, the first author's name, ethnicity, country, genotyping method, matching criteria, genotype number, and total number of cases and controls should be listed in the data table.

### Statistical analyses

Six genetic models as the allelic (T allele distribution frequency), recessive (TT vs. CC+CT), dominant (CC vs. TT+CT), homozygous (TT vs. CC), heterozygous (CT vs. CC), and additive (T vs. C) genetic models were adopted in the present meta-analysis. The odds ratios (ORs) and their corresponding 95% confidence intervals (CIs) were used to compare the association of *TGF-β1* gene -509C/T polymorphism and CAD. The heterogeneity among the studies was calculated by Chi-square-based Q-tests and the significance was set at *P* < 0.05 level (Cochran, 1968). If heterogeneity existed among the individual studies, the random-effect model (DerSimonian and Laird method) would be used (Dersimonian and Laird, 2015). Or else, the fixed-effect model was adopted (the Mantel–Haenszel method) (Mantel and Haenszel, 1959). The pooled OR was assessed by Z test with significance set at *P* < 0.05 level.

The HWE was evaluated by Fisher's exact test with significance set at *P* < 0.05 level. The potential publication bias was assessed by funnel plot. The funnel plot symmetry was evaluated by using the Egger's linear regression test on the natural logarithm scale of the OR with significance set at *P* < 0.05 level (Egger et al., 1997). The statistical analyses were performed by using Revman 5.0 and Stata 12.0 software (StataCorp, College Station, TX, USA).

## Results

### Studies and populations

All of information was abstracted from 6,377 CAD cases and 5,324 controls (Table 1) (Cambien et al., 1996; Syrris et al., 1998; Koch et al., 2006; Crobu et al., 2008; Drenos et al., 2009; Sudomoina et al., 2010; Zou et al., 2013; Pei et al., 2014). Sixteen manuscripts were found out by the retrieval process, among which eight papers conformed to the inclusion criteria. Among the eight excluded studies, three papers were of reviews character, and two manuscripts violated the HWE (Sie et al., 2006; Najar et al., 2011). Three papers were not involved with *TGF-β1* gene -509C/T polymorphism or CAD (Supplement S2). Six countries were included in the present meta-analysis as China, Italia, German, United Kingdom, France, and Russia. They belong to Chinese and Caucasian subgroups respectively. Two individual studies were included in the Chinese subgroup and six individual studies were included in the Caucasian subgroup.

**Table 1:** **Characteristics of the investigated studies of the association between the ***TGF-**β**1*** gene -509C/T polymorphism and CAD**.

**Author**	**Year**	**Country**	**Ethnicity**	**Genotype**	**CAD**			**Control**			**Matching criteria**	**sample size (CAD/control)**
					**CC**	**CT**	**TT**	**CC**	**CT**	**TT**		
Zou SM	2013	China	Han	PCR-LDR	49	153	138	49	128	94	Age, ethnicity,HDL-C	340/271
Pei F	2014	China	Han	PCR-RFLP	97	217	143	111	201	101	Age, sex, ethnicity, smoker, HP	457/413
Crobu F	2008	Italia	Caucasian	PCR-RFLP	67	87	47	80	92	29	Ethnicity	201/201
Koch W	2006	German	Caucasian	PCR-RFLP	1581	1659	417	564	508	139	Ethnicity	3657/1211
Syrris P	1998	UK	Caucasian	PCR-SSCP	301	284	70	124	97	23	Age, ethnicity	655/244
Cambien F	1996	FR,NIE	Caucasian	PCR-SSCP	240	257	66	263	297	69	Ethnicity	563/629
Sudomoina MA	2010	Russia	Caucasian	PCR-RFLP	77	150	37	90	103	19	Ethnicity	264/212
Drenos F	2009	England	Caucasian	PCR-RFLP	120	100	20	1090	885	168	Ethnicity	240/2143

*CAD, coronary artery disease; HDL-C, high density lipoprotein-cholesterol; HP, hypertension; UK, United Kingdom; FR, France; NIE, Northern Ireland. PCR-LDR, Polymerase chain reaction-ligase detection reaction; PCR-RFLP, Polymerase chain reaction-restriction fragment length polymorphism; PCR-SSCP, Polymerase Chain Reaction-Single Strand Conformation Polymerase; Case-control study design were adopted in the above studies*.

### Pooled analyses

A significant association between *TGF-β1* gene -509C/T polymorphism and CAD was detected in the total population under allelic (OR: 1.130, 95% CI: 1.060–1.200, *P* = 0.0001), recessive (OR: 1.390, 95% CI: 1.100–1.750, *P* = 0.006), dominant (OR: 0.857, 95% CI: 0.785–0.935, *P* = 2.507 × 10^−4^), homozygous (OR: 1.258, 95% CI: 1.098–1.442, *P* = 0.001), heterozygous (OR: 1.147, 95% CI: 1.046–1.257, *P* = 0.003), and additive genetic models (OR: 1.131, 95% CI: 1.063–1.204, *P* = 5.442 × 10^−5^).

In the subgroup analysis, there was a significant association between them in Chinese population under all of the genetic models (*P* < 0.05), except under the heterozygous genetic model (*P* >0.05).

In the Caucasian subgroup, a significant association between them was also detected under all of the genetic models (*P* < 0.05), except under the recessive genetic model (*P* > 0.05).

In the subgroup analysis, there was a significant association between them in Chinese population under allelic (OR: 1.260, 95% CI: 1.090–1.460, *P* = 0.005), recessive (OR: 1.800, 95% CI: 1.400–2.330, *P* = 3.241 × 10^−6^), dominant (OR: 0.743, 95% CI: 0.577-0.957, *P* = 0.022), homozygous (OR: 1.561, 95% CI: 1.164–2.092, *P* = 0.003), and additive genetic models (OR: 1.259, 95% CI: 1.088–1.458, *P* = 0.002). However, no significant association between *TGF-β1* gene -509C/T polymorphism and CAD was found under heterozygous genetic model (OR: 1.222, 95% CI: 0.933–1.600, *P* = 0.146).

**Figure 1:**
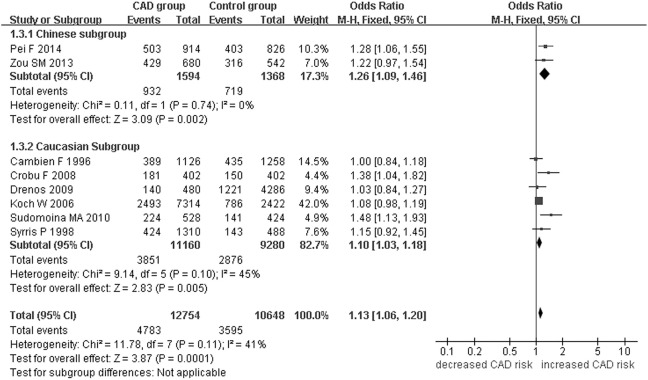
**Forest plot of CAD associated with ***TGF-β1*** gene -509C/T polymorphism under an allelic genetic model (distribution of T allelic frequency of ***TGF-β1*** gene -509C/T polymorphism)**.

**Figure 2:**
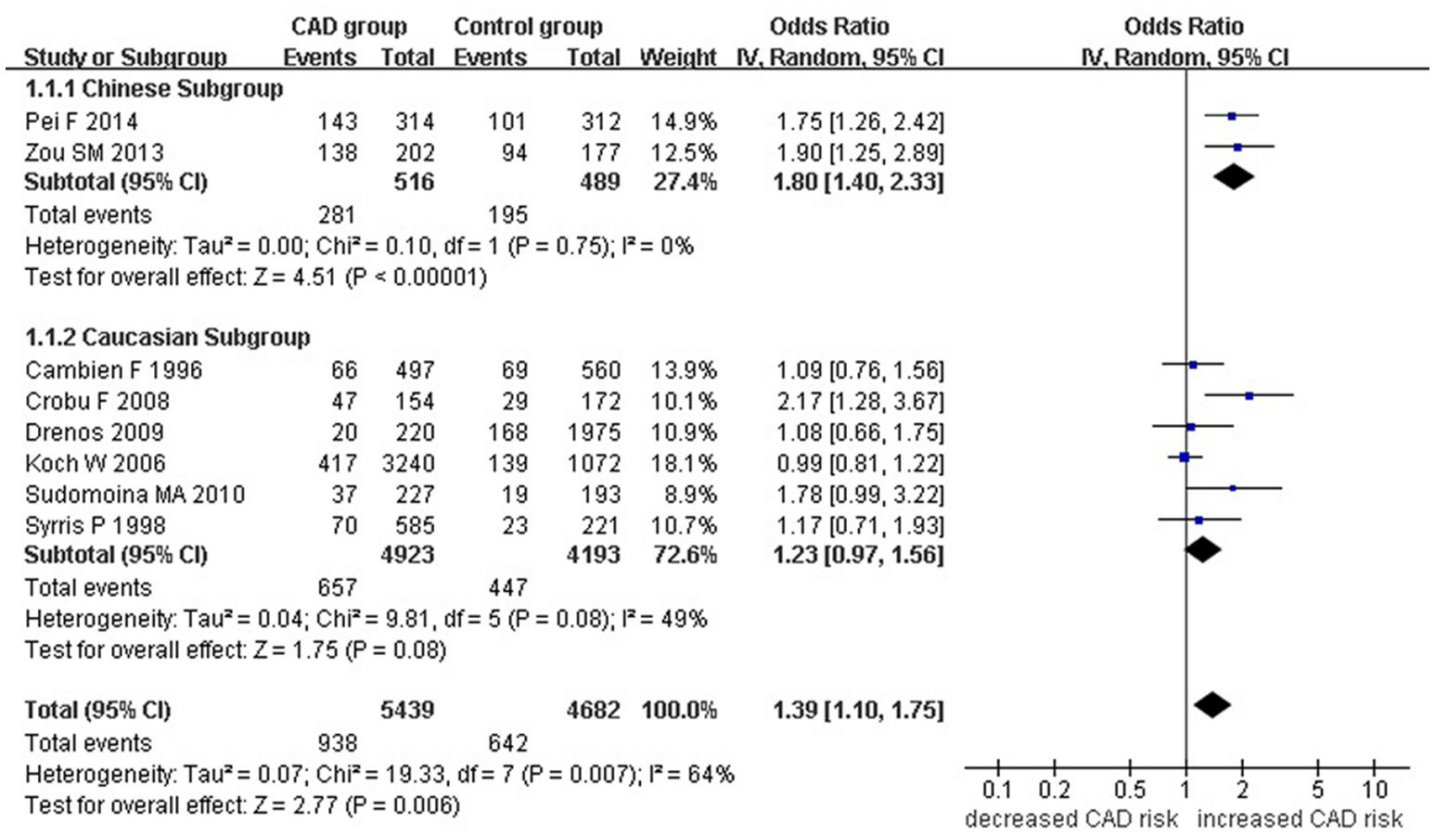
**Forest plot of CAD associated with ***TGF-β1*** gene -509C/T polymorphism under a recessive genetic model (TT vs. CC+CT)**.

**Figure 3:**
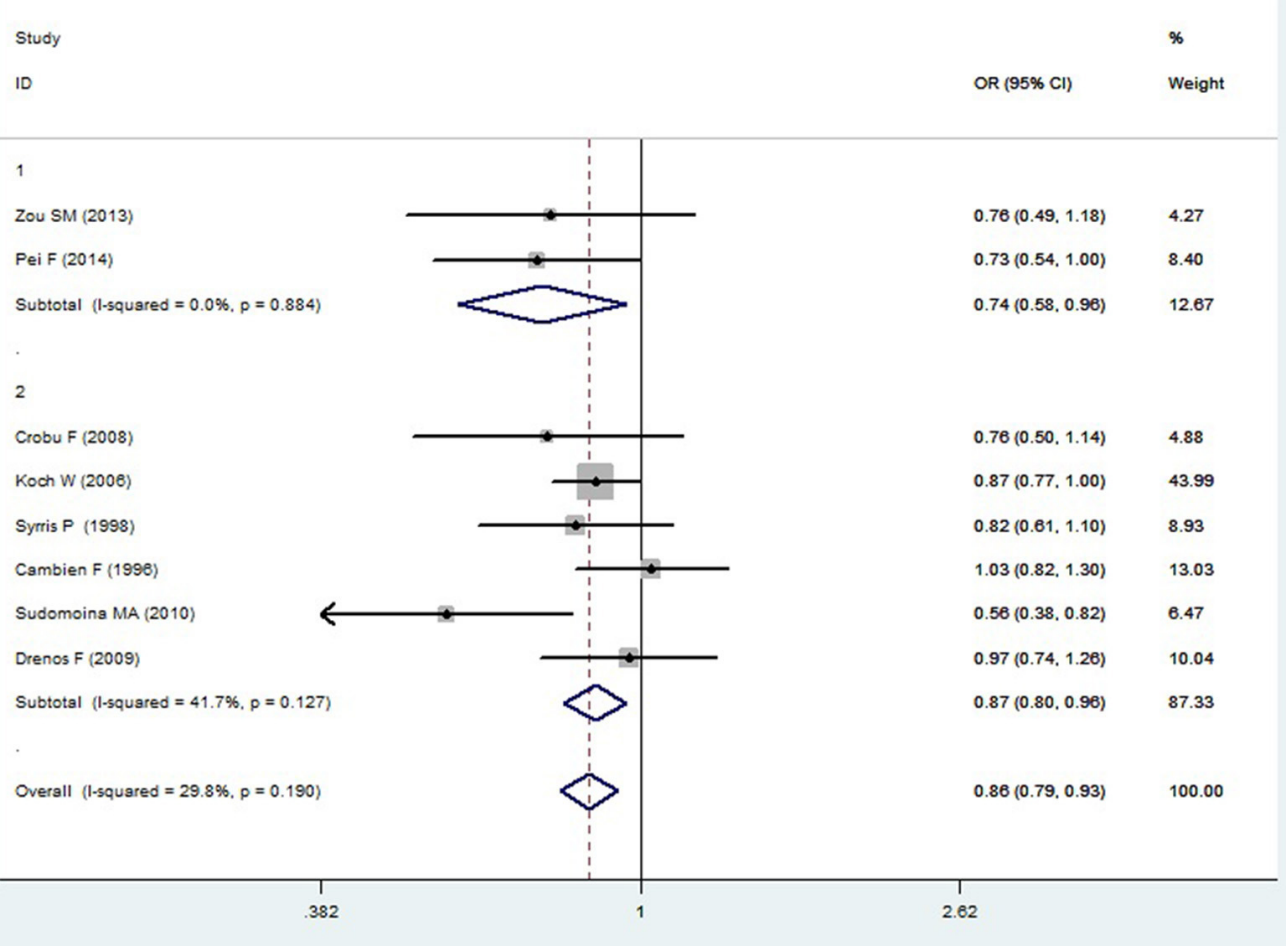
**Forest plot of CAD associated with ***TGF-β1*** gene -509C/T polymorphism under a dominant genetic model (CC vs. TT+CT)**.

**Figure 4:**
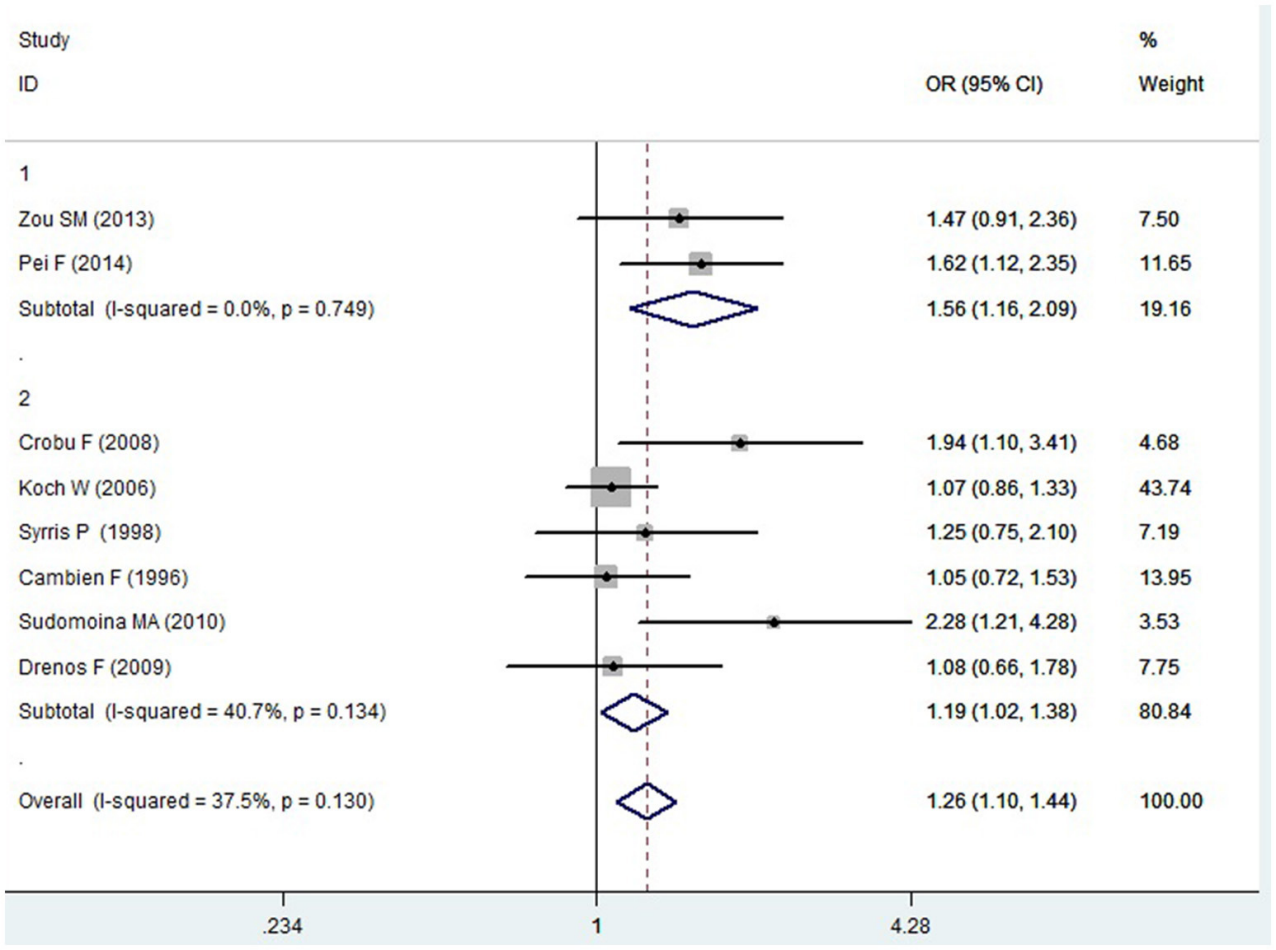
**Forest plot of CAD associated with ***TGF-β1*** gene -509C/T polymorphism under a homozygous genetic model (TT vs. CC)**.

**Figure 5:**
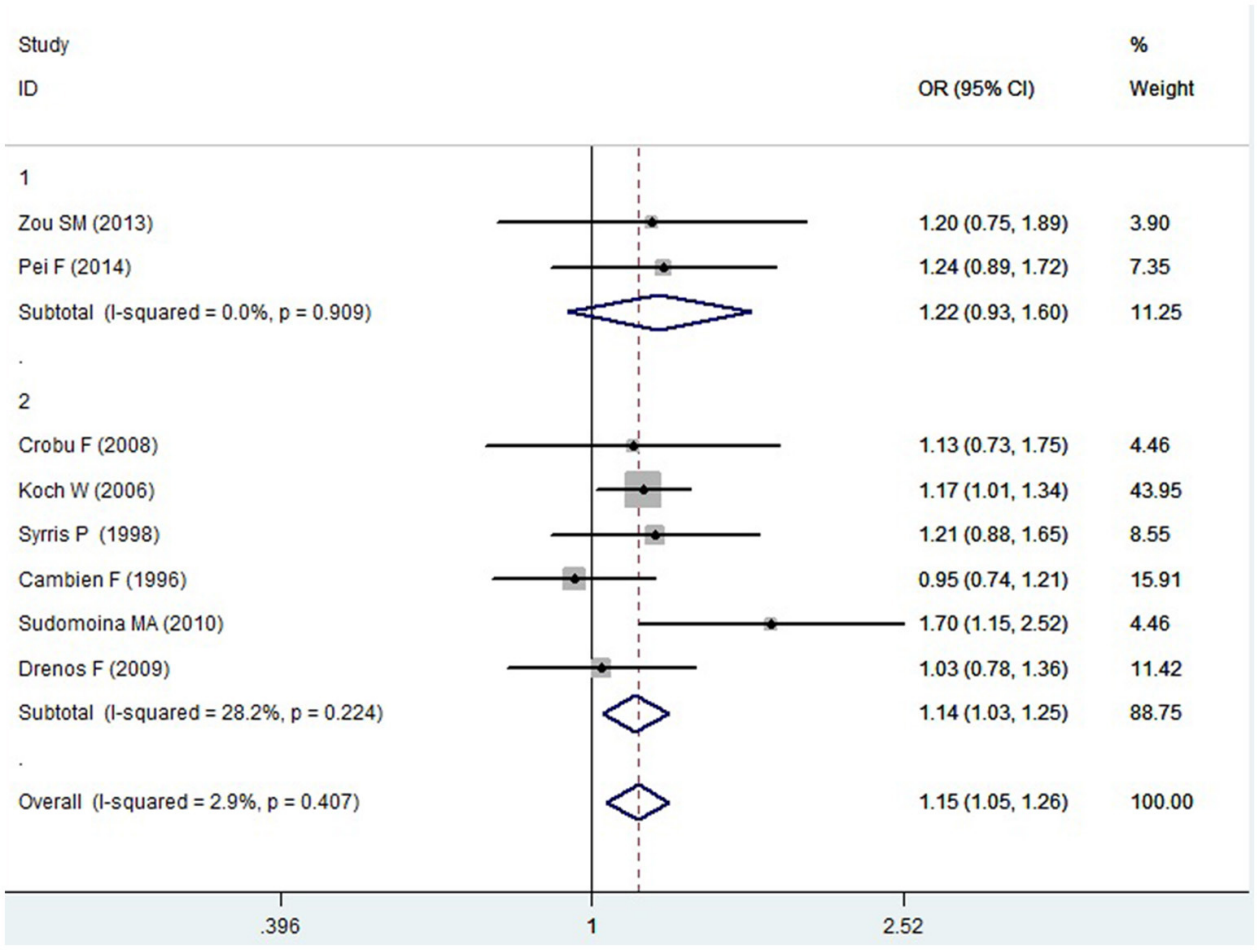
**Forest plot of CAD associated with ***TGF-β1*** gene -509C/T polymorphism under a heterozygous genetic model (CT vs. CC)**.

**Figure 6:**
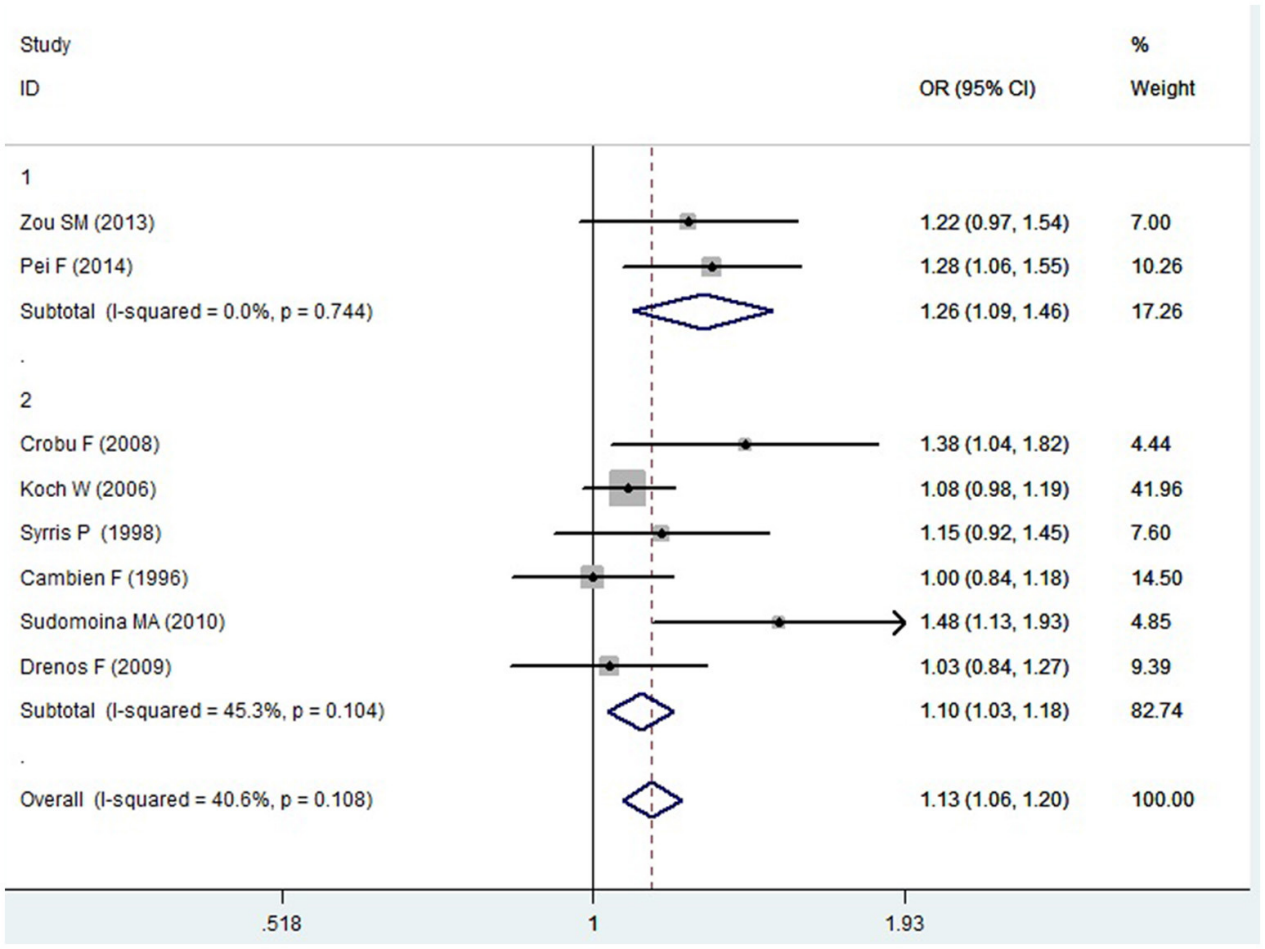
**Forest plot of CAD associated with ***TGF-β1*** gene -509C/T polymorphism under an additive genetic model (T vs. C)**.

In the Caucasian subgroup, a significant association between them was also detected under allelic (OR: 1.100, 95% CI: 1.030–1.180, *P* = 0.002), dominant (OR: 0.873, 95% CI: 0.796-0.958, *P* = 0.004), homozygous (OR: 1.187, 95% CI: 1.017–1.384, *P* = 0.029), heterozygous (OR: 1.137, 95% CI: 1.031–1.254, *P* = 0.010), and additive genetic models (OR: 1.105, 95% CI: 1.031–1.184, *P* = 0.005). However, no significant association was detected under recessive genetic model (OR: 1.230, 95% CI: 0.970–1.560, *P* = 0.08) (Table 2, Figures 1–6).

**Table 2:** **Summary of meta-analysis of association between the ***TGF-**β**1*** gene -509C/T polymorphism and CAD**.

**Genetic model**	**Pooled OR (95% CI)**	***Z*****-value**	***P*****-value**	**Study number**	**CAD size**	**Control size**	***P***_**heterogeneity(***I***^2^%)**_
Allelic genetic model	1.130 (1.060–1.200)	3.87	0.0001^*^	8	6377	5324	0.11 (41.0)
Chinese subgroup	1.260 (1.090–1.460)	2.83	0.005^*^	2	797	684	0.74 (0)
Caucasian subgroup	1.100 (1.030–1.180)	3.09	0.002^*^	6	5580	4640	0.10 (45.0)
Recessive genetic model	1.390 (1.100–1.750)	2.77	0.006^*^	8	6377	5324	0.007^*^ (64.0)
Chinese subgroup	1.800 (1.400–2.330)	4.51	3.241 × 10^−6^^*^	2	797	684	0.75 (0)
Caucasian subgroup	1.230 (0.970–1.560)	1.75	0.08	6	5580	4640	0.08 (49.0)
Dominant genetic model	0.857 (0.785–0.935)	3.48	2.507 × 10^−4^^*^	8	6377	5324	0.190 (29.8)
Chinese subgroup	0.743 (0.577–0.957)	2.30	0.022^*^	2	797	684	0.884 (0)
Caucasian subgroup	0.873 (0.796–0.958)	2.87	0.004^*^	6	5580	4640	0.127 (41.7)
Homozygous genetic model	1.258 (1.098–1.442)	3.30	0.001^*^	8	6377	5324	0.130 (37.5)
Chinese subgroup	1.561 (1.164–2.092)	2.98	0.003^*^	2	797	684	0.749 (0)
Caucasian subgroup	1.187 (1.017–1.384)	2.18	0.029^*^	6	5580	4640	0.134 (40.7)
Heterozygous genetic model	1.147 (1.046–1.257)	2.92	0.003^*^	8	6377	5324	0.407 (2.9)
Chinese subgroup	1.222 (0.933–1.600)	1.45	0.146	2	797	684	0.909 (0)
Caucasian subgroup	1.137 (1.031–1.254)	2.58	0.010^*^	6	5580	4640	0.224 (28.2)
Additive genetic model	1.131 (1.063–1.204)	3.87	5.442 × 10^−5^^*^	8	6377	5324	0.108 (40.6)
Chinese subgroup	1.259 (1.088–1.458)	3.09	0.002^*^	2	797	684	0.744 (0)
Caucasian subgroup	1.105 (1.031–1.184)	2.83	0.005^*^	6	5580	4640	0.104 (45.3)

* *P ≤ 0.05. CAD, coronary artery disease; CI, confidence interval; OR, odds ratio; CAD size, the total number of CAD cases; control size, the total number of control group; Allelic genetic model, T allele distribution frequency; recessive genetic model: TT vs. CC+CT, Dominant genetic model: CC vs. TT+CT; Homozygous genetic mode, TT vs. CC; Heterozygous genetic model, CT vs. CC; Additive genetic model, total T allele vs. total C allele*.

There was no significant heterogeneity in the whole population under all of the genetic models (*P*_heterogeneity_ > 0.05) except under the recessive genetic model (*P*_heterogeneity_ < 0.05). In the subgroup analysis stratified by ethnicity, no heterogeneity was detected either in the Chinese subgroup or in the Caucasian subgroup under the recessive genetic model (*P*_heterogeneity_ > 0.05).

### Bias diagnostics

**Figure 7:**
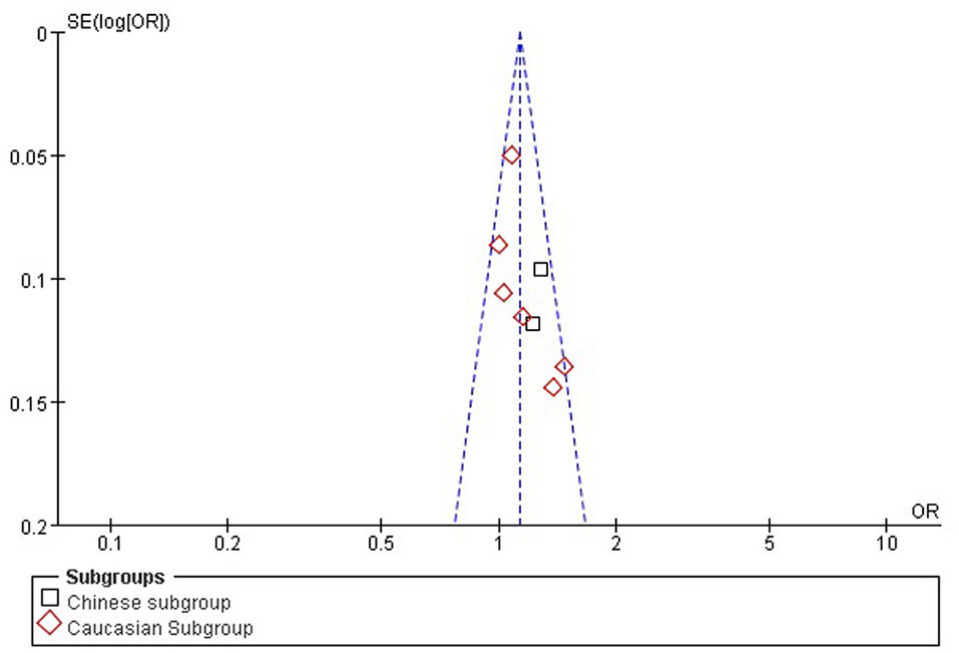
**The funnel plot for studies of the association of CAD associated with ***TGF-β1*** gene -509C/T polymorphism under an allelic genetic model (distribution of T allelic frequency of ***TGF-β1*** gene -509C/T polymorphism)**. The horizontal and vertical axis correspond to the OR and confidence limits. OR, odds ratio, SE; standard error.

The publication bias among the individual studies was evaluated by using the funnel plot and Egger's test. No publication bias was visualized in the funnel plot under the allelic genetic model (Figure 7). Additionally, no significant difference was detected in the Egger's test yet, which indicated that there was no publication bias in the current meta-analysis by using allelic genetic model (*T* = 2.04, *P* = 0.088).

## Discussion

In the current meta-analysis, a significant association was detected in the whole population between *TGF-β1* gene -509C/T polymorphism and CAD under allelic (OR: 1.130), recessive (OR: 1.390), dominant (OR: 0.857), homozygous (OR: 1.258), heterozygous (OR: 1.147), and additive genetic models (OR: 1.131). In the subgroup analysis by ethnicity, a significant association in Chinese subgroup was also found under allelic (OR: 1.260), recessive (OR: 1.800), dominant (OR: 0.743), homozygous (OR: 1.561), and additive genetic models (OR: 1.259). A significant association was also found in the Caucasian subgroup under allelic (OR: 1.100), recessive (OR: 1.230), dominant (OR: 0.873), homozygous (OR: 1.187), heterozygous (OR: 1.137), and additive genetic models (OR: 1.105). In conclusion, it was implied that the carriers with T allele of *TGF-β1* gene -509C/T polymorphism might increase CAD risk, both in the Chinese and Caucasian population.

No significant heterogeneity was detected in the whole population under all of the genetic models except the recessive genetic model. Under the recessive genetic model, a significant heterogeneity was detected in the whole population (*P*_heterogeneity_ = 0.007). In the subsequent subgroup analysis stratified by ethnicity, the heterogeneity did not exist any longer in the Chinese or Caucasian subgroup (*P* > 0.05).

The pathogenesis of CAD include coronary atherosclerosis, embolism, dissecting aneurysm, coronaritis, malformation of coronary artery, metabolic disease, infection, trauma and et al, of which coronary atherosclerosis accounts for more than 90%. Atherosclerosis is proliferative inflammatory lesions. The pathological changes include the vascular endothelial cells dysfunction, the vascular smooth muscle cells (VSMCs) proliferation and migration, the extracelluar matrix proliferation, the inflammatory cells infiltration, the fibrous cap formation, and rupture and angiogenesis (Yang et al., 2006).

Recently the researches about the association of some cytokines and CAD have become the research hot, particularly the TGF-β1. TGF-β1 is the disulfide bonded dimer which consists of two 12.5 kDa polypeptide chains. The newly synthesized TGF-β1 is the inactive precursor protein which is constituted with 391 amino acid residues. The 25 kDa active TGF-β1 is generated by the enzymolysis *in vivo* or disposed by acid, urea and protease *in vitro*. TGF-β1 plays the multifunctional regulation role as inhibiting the inflammatory cytokines, regulating the cells proliferation and differentiation, embryonic development, damage repair and angiogenesis through the endocrine, and paracrine mechanisms (Feinberg et al., 2000).

TGF-β1 plays the important roles in the coronary atherosclerosis. TGF-β1 could down regulate many cytokines level and function. It can inhibit TNF-α synthesis and reduce its effect. TGF-β1 can decrease the VCAM-1 expression induced by the inflammatory cytokines and inhibit the leukocytes adhering to the endothelial cells (Agassandian et al., 2015). TGF-β1 can decrease the IFN-γ effect. TGF-β1 can reduce the macrophage activity by decreasing the IL-1 activity (Ruscetti et al., 1992; Gamble et al., 1993; Park et al., 2000). TGF-β1 mainly has 4 kinds of functions. (1) TGF-β1 inhibits the VSMCs migration and proliferation; (2) TGF-β1 induces the apoptosis of leukocytes which participates in the vascular injury; (3) TGF-β1 reduces the adherence of the inflammatory cells to the endothelial cells; (4) TGF-β1 helps the vascular protection by inhibiting the human metalloenzyme and unstability reaction in the instable plaque (Wahl et al., 1989; Mccaffrey et al., 1999; Blobe et al., 2000). *TGF-β1* gene -509C/T mutation might decrease the affinity of the promoter and transcription factor, reduce TGF-β1 transcription level and promote the coronary atherosclerosis.

In 2012, Morris et al. performed a meta-analysis on the association of CAD with *TGF-β1* gene -509C/T polymorphism. In this meta-analysis, they concluded that T alleles of rs1800469 in *TGF-β1* were associated with complications of CAD (Morris et al., 2012). However, only four individual studies were included. In 2012, Lu et al. performed another meta-analysis on the association of CAD with *TGF-β1* gene -509C/T polymorphism. They found that T allele carriers of rs1800469 in *TGF-β1* have a 15% increased risk of CAD. In their meta-analysis, only seven individual studies were included. Among these included individual studies, one study by Sie deviating from the HWE was not excluded (Lu et al., 2012). Hence, the results from the two above meta-analysises published before were not as accurate as that deduced from the current meta-analysis.

However, there were still some limitations in the present meta-analysis. The current meta-analysis is still short of prospective or large-scale studies on the association of *TGF-β1* gene -509C/T polymorphism and CAD. The serum TGF-β1 level was influenced not only by the *TGF-β1* gene -509C/T polymorphism, but also by other polymorphism as -800 G/A, 868 T/C, 913 G/C, and 11929 C/T gene polymorphism. Many other environmental factors as age, smoke, gender, obesity, dyslipidemia, diabetes mellitus, and hypertension might affect the CAD development. Since these factors were not matched in most of individual studies, it was difficult to analyze their effect on the CAD progress (Lu et al., 2012). Additionally, according to our knowledge it is >1000-fold confirmed association between given polymorphism and the risk of CAD. It might not influence our every day clinical practice concerning diagnosis or therapy yet. However, it can help us to screen out the susceptible population for CAD and prevent the CAD progression in advance. Therefore, the association of *TGF-β1* gene -509C/T polymorphism and CAD is important to the prevention of CAD in the high risk population.

As CAD is a polygenic disease, various micro-effect genes might have an effect on the CAD risk. Other genes polymorphisms as *CD14* gene −159C/T polymorphism, *Interleukin-6* C-572G polymorphism, *methylenetetrahydrofolate reductase* gene C677T polymorphism, *intercellular adhesion molecule-1* gene E469K polymorphism, *ATP-binding cassette transporter A1* gene R219K polymorphism, *apo A5* gene −1131T/C, *FgB* −455G/A, −148C/T, and *CETP* gene TaqIB polymorphisms, *plasminogen activator inhibitor-1* gene 4G/5G polymorphism might increase the CAD risk (Li, 2012a,b; Li et al., 2012, 2013, 2015a,b; Yanyan, 2012).

In short, *TGF-β1* gene -509C/T polymorphism was significantly associated with increased CAD risk both in the Chinese and Caucasian population. The persons with the T allele of *TGF-β1* gene -509C/T polymorphism might be predisposed to CAD risk. This conclusion might guide us to establish a novel CAD individualized treatment. In consideration of the above limitations, more researches on the association of *TGF-β1* gene -509C/T polymorphism and CAD needed to be conducted to further confirm the conclusions in the future.

## Author contributions

Conceived and designed the experiments: YL and YZ. Performed the experiments: YL and GG. Analyzed the data: YL and HG. Contributed reagents/material/analysis tools: YL and XY. Wrote the manuscript, reference collection, data management, statistical analyses, paper writing, and study design: YL.

### Conflict of interest statement

The authors declare that the research was conducted in the absence of any commercial or financial relationships that could be construed as a potential conflict of interest.
