# Supplementary material for: TGF-β1 Gene -509C/T Polymorphism and Coronary Artery Disease: An Updated Meta-Analysis Involving 11,701 Subjects
Source: Front Physiol. 2017 Feb 23;8:108. doi: 10.3389/fphys.2017.00108 (PMC5322195; doi:10.3389/fphys.2017.00108)
Supplement: Supplementary file 2 [file Table2.DOCX]

**PRISMA 2009 Flow Diagram**


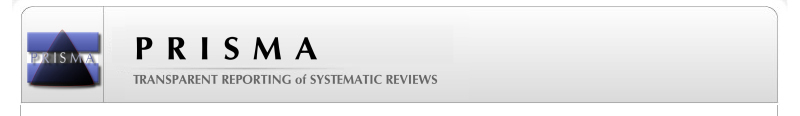


Studies included in qualitative synthesis
(n =8)

Records excluded for no association with *TGF-β1* gene -509C/T polymorphism or CAD

(n =3)

Records excluded for repeated publication
(n = 0 )

Full-text articles excluded for deviation from HWE (n =2 )

Records excluded for review characteristic
(n =3 )

Full-text articles assessed for eligibility
(n =11)

Records screened
(n =13 )

Records after duplicates removed
(n =16)

Additional records identified through other sources
(n =0 )

## Identification

## Eligibility

## Included

## Screening

Records identified through database searching
(n =16 )
